# Measuring Thiaminase Activity in Fish Extracts using Fluorescence Spectrophotometry

**DOI:** 10.1021/acsmeasuresciau.5c00091

**Published:** 2025-09-19

**Authors:** Drew Porter, Cody Pinger

**Affiliations:** † College of Fisheries and Ocean Sciences, University of Alaska Fairbanks, 17101 Pt. Lena Loop Road, Juneau, Alaska 99801, United States; ‡ NOAA, National Marine Fisheries Service, Alaska Fisheries Science Center, Auke Bay Laboratories, 17109 Pt. Lena Loop Road, Juneau, Alaska 99801, United States

**Keywords:** thiaminase, thiamine deficiency, fluorescence spectrophotometry, forage fishes, enzyme kinetics, pH optimum, Bering Sea

## Abstract

Thiaminase is an enzyme that destroys thiamine (vitamin B1) and is present in various fishes, mussels, plants, and bacteria. A sensitive and versatile assay is needed to measure thiaminase activity in complex biological samples using common reagents and variable reaction conditions. We developed a simple assay that uses fluorescence spectrophotometry and a microplate reader to measure thiaminase activity in fish tissue extracts via the destruction of added thiamine over time. Thiamine concentration, cosubstrate choice and concentration, and pH can be varied according to the needs of the investigator. Using this assay, we successfully measured common enzyme kinetic constants for thiaminase from a Pacific herring extract. The enzyme exhibited Michaelis–Menten kinetics (*V*_max_ = 13.8 nmol T g^–1^ min^–1^, *K*_m_ = 27.4 μM) with respect to thiamine and showed positive cooperativity for nicotinic acid as a cosubstrate (Hill equation, *V*_max_ = 12.1 nmol T g^–1^ min^–1^, *K*_half_ = 20.3 mM, *n* = 3.2). The thiaminase pH optimum of a rainbow smelt extract was also successfully measured (pH = 5.06 ± 0.06). Thiaminase activities of common forage fishes from the northern Bering Sea (2024) were compared using this fluorescence-based assay and the conventional 4-nitrothiophenol assay, marking the first reported measurements of thiaminase activity in this ecosystem. This new fluorescence-based assay is a tool that can be used for studying thiaminase dynamics in specimens under a variety of reaction conditions, enabling studies that will improve the understanding of factors driving thiamine deficiency in consumers.

## Graphical abstract

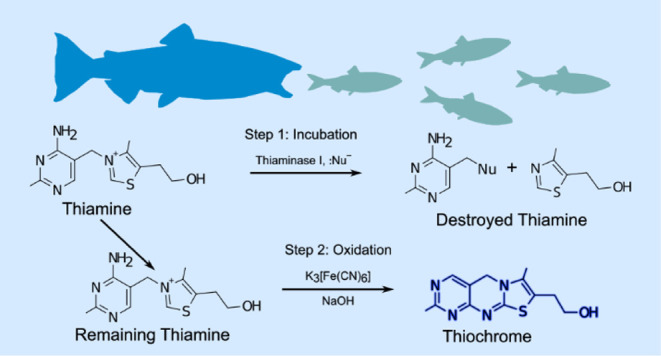

## Introduction

Thiamine (vitamin B1) is an essential dietary nutrient for all multicellular animals and a requirement for a range of biochemical functions including cellular respiration. Thiamine deficiency has been implicated as a cause of widespread population declines in a variety of species and ecosystems worldwide.^1–9^ In fish, thiamine deficiency is known to alter life-history through neurological impairment, reduced growth, reproductive failure, and early life mortality. Thiamine deficiency has been documented in several fish species, including Atlantic salmon (*Salmo salar*) in the Baltic Sea and New York’s Finger Lakes, steelhead (*Oncorhynchus mykiss*) in Oregon, and several salmonid species in the Laurentian Great Lakes.^2,5,10–13^ Recently, studies have also identified potential thiamine deficiency in Chinook salmon (*O. tshawytscha*) populations from the Yukon River in Alaska and the Sacramento River in California.^9,14,15^ Though thiamine deficiency in fish has been studied for more than 50 years, the causes and underlying mechanisms are not well understood.

Thiamine deficiency may result from various dietary factors, such as limited thiamine intake or high lipid and polyunsaturated fatty acid content, but is frequently associated with consumption of thiaminase I-rich prey.^16–22^ Thiaminase I (hereafter thiaminase) is an enzyme detected in several species of bacteria, fish, shellfish, and ferns that destroys thiamine by cleaving the pyrimidine and thiazole components.^23^ Though the physiological function of thiaminase is unresolved, it is known to be produced by bacteria, and *de novo* synthesis in fish was recently confirmed.^24,25^ Thiamine deficiency in animals caused by diets containing raw fish was reported in the early 1930s.^26–28^ The presence of a thiamine destroying component in fish, later termed thiaminase, was confirmed using qualitative chemistry techniques in 1941.^29^ Many subsequent studies have examined the presence and activity of thiaminase in prey species. Thiaminase-rich fishes such as alewife (*Alosa pseudoharengus*) in the Laurentian Great Lakes and New York’s Finger Lakes, Atlantic herring (*Clupea harengus*) in the Baltic Sea, and northern anchovy (*Engraulis mordax*) in the Pacific Ocean have all been hypothesized to cause thiamine deficiency in salmonid consumers.^3,6,9,15,19,20^

Precise analytical tools are needed to investigate sources of thiaminase and potential causes of thiamine deficiency. Current methods used for quantifying thiaminase activity in fishes are the spectrophotometric 4-nitrothiophenol assay (4-NTP) and the radiometric ^14^C-thiamine assay.^30–34^ Both methods have strengths and limitations. When paired with a microplate reader, the 4-NTP assay is a valuable high throughput screening tool, allowing rapid generation of thiaminase activity data for the large sample sizes required for ecosystem studies. The assay indirectly measures thiaminase activity by measuring the absorbance change at 411 nm over a period of time. This wavelength measures the cosubstrate 4-NTP and assumes a 1:1 mol ratio of 4-NTP and thiamine breakdown by the thiaminase enzyme. The molar extinction coefficient of 4-NTP is dependent on pH, limiting the assay to a relatively high pH range (approximately >6.9) and preventing studies at the low pH ranges found in the gut of consumers (e.g., Atlantic salmon stomach chyme may range from pH 3.5 to 4.8 during digestion).^35,36^ Additionally, studies are limited to using 4-NTP as the cosubstrate, preventing investigation of different cofactors that may occur in nature (e.g., nicotinic acid). The radiometric assay is dependent on radioactive [^14^C-thiazole]-thiamine and the measurement of the liberated breakdown product (thiazole) after extraction in ethyl acetate. Though the assay is highly sensitive, relatively rapid, and adaptable to different pH and cosubstrates, [^14^C-thiazole]-thiamine is not currently commercially available and must be custom synthesized, a process that can be financially prohibitive. Further, the laboratory instrumentation necessary for detection is relatively uncommon, thus restricting the assay’s accessibility.

Fluorescence spectrophotometry is an established method for measuring thiamine (via fluorescent thiochrome), and has been used to measure its destruction in the presence of thiaminase to estimate enzyme activity rates. An early fluorescence-based assay described measuring thiaminase activity in fish extracts using large sample masses (20 g) and extract volumes (180 mL) and a traditional cuvette-based spectrofluorometer.^37^ Similarly, a fluorescence-based assay has been used recently to measure thiaminase activity in several strains of bacteria.^32^ However, these bacterial samples represent relatively simple matrices compared to complex samples such as whole fish tissue extracts.

Here, we describe a method for measuring thiaminase activity in whole fish extracts by measuring the destruction of added thiamine over time using fluorescence spectrophotometry and conversion of thiamine to fluorescent thiochrome. This method has been iteratively developed to improve its sensitivity, minimize sample volumes and matrix interferences, and increase its throughput and versatility. The method utilizes a microwell plate reader and is validated by high performance liquid chromatography (HPLC) measurements. Importantly, we demonstrate the ability to perform qualitative studies on thiaminase enzyme dynamics, including Michaelis–Menten kinetics, cosubstrate investigations, and pH optima analyses. Further, thiaminase values from common prey fish collected from the northern Bering Sea in 2024 are reported along with intermethod comparison to 4-NTP data, providing insight into previous reports of thiamine deficiency in consumers in this ecosystem.

## Materials and Methods

### Materials

Thiamine hydrochloride (98.5–101.5%), nicotinic acid (99%), and 4-nitrothiophenol (96%) were purchased from Thermo Scientific Chemicals. Dimethyl sulfoxide (≥99.9%, ACS grade) and trichloroacetic acid (TCA; 95.0–105.0%) were obtained from VWR Chemicals. Anhydrous monobasic sodium phosphate (≥99%) and sodium hydroxide (≥97.0%) were sourced from Fisher BioReagents. Anhydrous dibasic sodium phosphate (≥99%, ACS grade) and sodium chloride (≥99.0%, ACS grade) were acquired from Fisher Chemical. Potassium ferricyanide (≥99.0%, ACS grade) was supplied by LabChem, and Tris­(2-carboxyethyl) phosphine hydrochloride was purchased from Soltec Ventures. All buffers and reagents were prepared using ultrapure water (resistivity ≥18.2 MΩ·cm). Standard Reference Material 1946 (SRM 1946) lake trout muscle tissue was purchased from the National Institute of Standards and Technology (Gaithersburg, MD).^38^

### Sample Collection

Initial experiments used Pacific herring (*Clupea pallasii*) collected from the Gulf of Alaska. All other fish used in this study were opportunistically sampled during National Marine Fisheries Service ecosystem monitoring surveys in the northern Bering Sea in 2024 under NOAA Scientific Research Permit # 2024-5. Rainbow smelt (*Osmerus mordax*), Pacific herring, Alaska walleye pollock (*Gadus chalcogrammus*), sand lance (*Ammodytes hexapterus*), capelin (*Mallotus villosus*), and saffron cod (*Eleginus gracilis*) were collected via trawl net, immediately frozen at −20 °C, shipped frozen to Auke Bay Laboratories (Juneau, Alaska), and stored at −80 °C until processing and analysis.

### Fish Tissue Preparation and Extraction

Prior to analysis of thiaminase activity, whole-fish samples were cryogenically homogenized to a fine powder using liquid nitrogen and a Pulverisette 2 mortar grinder (Fritsch, Pittsboro, NC), ensuring samples remained frozen throughout. Whole-fish extracts were prepared by combining tissue homogenates with phosphate buffer (50 mM phosphate, 100 mM NaCl, pH 6.5) at a ratio of 1 g tissue to 2.5 mL buffer (2:5, w:v) in low-retention microcentrifuge tubes (Thermo Scientific), following Zajicek et al.^33^ Samples were vortexed, centrifuged (21,000*g*, 4 °C, 10 min), and filtered through 30 μm pore polyethylene spin columns (Pierce, Thermo Scientific). Filtered supernatants were aliquoted into 4–8 replicate microcentrifuge tubes (200 μL each) and stored at −80 °C until the day of analysis.

### Thiaminase Fluorescence Method

#### Method Overview

In brief, fish extracts were mixed with buffered nicotinic acid (i.e., the nucleophilic cosubstrate) and a known quantity of thiamine in microcentrifuge tubes and incubated at 37 °C to allow thiaminase in the fish extract to react with added thiamine. The reaction was stopped after a set incubation time (0, 10, and/or 30 min) and the remaining thiamine was quantified, allowing calculation of the rate at which it was destroyed by the thiaminase enzyme present in the fish extract. A mechanistic diagram of the method is provided in Figure 1.

**Figure 1:**
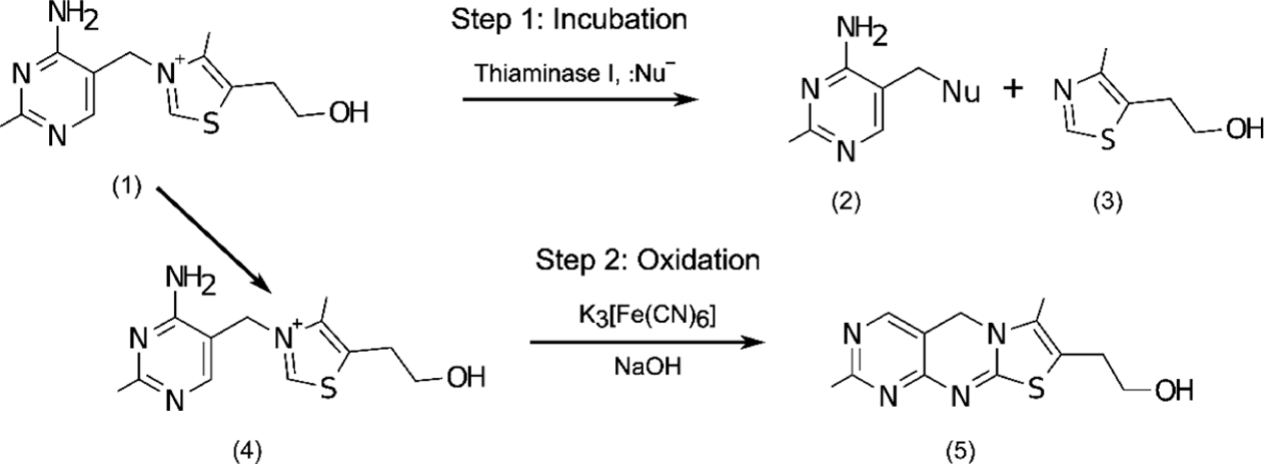
Mechanistic diagram of the fluorescence-based assay. During the incubation step thiamine (1) is cleaved by thiaminase into pyrimidine (2) and thiazole (3) fragments. During the oxidation step, remaining undestroyed thiamine (4) in the sample is converted to fluorescent thiochrome (5) for measurement.

#### Solution and Sample Preparation

All assay steps were carried out in low-retention 1.5 mL microcentrifuge tubes (Thermo Scientific). An 80 mM nicotinic acid solution was prepared in buffer (50 mM phosphate, 100 mM NaCl, pH 6.5). A 1 mM thiamine HCl stock solution was prepared in ultrapure water (resistivity ≥18.2 MΩ·cm), aliquoted (1 mL), and stored at −80 °C. A 3% (w/v) TCA solution was prepared and stored at 4 °C.

Prior to analysis, fish extracts and a thiamine aliquot were removed from the freezer and thawed at room temperature. The thiamine stock was diluted to 400 μM with ultrapure water, and a 1 mL aliquot of nicotinic acid solution was preheated to 37 °C for 10 min.

#### Thiaminase Sample Reaction

Unless noted otherwise, each reaction mix was prepared to contain 25 μL of fish extract, 25 μL of 400 μM thiamine HCl, and 50 μL of 80 mM nicotinic acid, yielding final reaction concentrations of 100 μM thiamine HCl and 40 mM nicotinic acid, and measuring pH 4.4. Initial trial experiments contained 25 μM thiamine and 30 mM nicotinic acid. Enzyme kinetics assays informed the increase of nicotinic acid to 40 mM and thiamine to 100 μM to provide maximal activity.

To prepare the initial time point samples, 50 μL preheated nicotinic acid solution was added to 25 μL fish extract followed by addition of 25 μL thiamine solution. Immediately, 800 μL of 3% TCA was added to quench the reaction, and the exact time elapsed was recorded. The same steps were followed for the incubated samples, but the reaction mixture was incubated for 10 or 30 min at 37 °C before quenching with TCA. Elapsed time was recorded for all samples. Quenched initial time point samples were held at 4 °C during the incubation step.

All reaction vials were then heated in a boiling water bath for 5 min to precipitate proteins, cooled on ice for 20 min, and then centrifuged (21,000*g*, 4 °C, 10 min), similar to the procedure described by Brown et al.^39^ Following centrifugation, 200 μL of each sample supernatant was diluted with 800 μL of ultrapure water in order to reduce matrix interferences and decrease the concentration of TCA, allowing for better conversion to thiochrome in the following step.

#### Thiochrome Preparation and Thiamine Quantitation

Thiamine was converted to fluorescent thiochrome by combining 425 μL of the diluted sample with 45 μL of 1 M sodium hydroxide and 30 μL of 3 mM potassium ferricyanide in a new microcentrifuge tube. After mixing, 100 μL of each mixture was transferred in triplicate to a black 96-well nonbinding surface microplate (Corning), and fluorescence was measured with a BioTek Synergy H1 Hybrid plate reader at excitation wavelength: 370 nm, emission wavelength: 430 nm. To quantify thiamine, fluorescence intensity values from triplicate wells were averaged and converted to molar amounts by comparison to an external standard calibration curve. These standards (0, 1.25, 2.5, 5, and 10 nmol thiamine) were prepared and treated the same as samples, albeit with buffer void of any fish tissue. A detailed description of calibration standard preparation is provided in the Supporting Information.

Thiaminase activity was calculated as shown in eq 1

1 $$A_{\mathrm{thiaminase}}=\frac{T_{\mathrm{initial}}-T_{\mathrm{final}}}{mt}[\mathrm{nmol T}\;g^{‐1\;}\min^{‐1}]$$

where *A*_thiaminase_ is the rate of thiamine destruction per gram of fish tissue (nmol T g^–1^ min^–1^), *T*_initial_ and *T*_final_ are the nmol thiamine measured in the initial and incubated samples, *m* is the mass of fish tissue in each sample (g), and *t* is incubation time (min).

Reported activity values represent the mean of duplicate measurements for each sample. Samples were initially tested using a 10 min incubation; samples with no detectable activity were reanalyzed using a 30 min incubation. All incubated samples were compared to measurements of initial time point samples (i.e., 0 min). Samples with a calculated thiaminase activity exceeding 150 nmol T g^–1^ min^–1^ were reanalyzed after diluting the extract 1:4 with phosphate buffer to prevent the reactions from becoming limited by thiamine.

#### Initial Experiments – HPLC and Plate Reader

Initial experiments used high-performance liquid chromatography (HPLC) with fluorescence detection (Supporting Information for instrument and details) and a microplate reader to measure thiamine (via thiochrome fluorescence) and track its destruction over time.^39^ The experiments were designed to determine if there were interfering fluorescence peaks present in the samples and if thiamine degraded in the absence of thiaminase under the described reaction conditions. Lake trout muscle (SRM 1946) extract was used as a suitable matrix blank (i.e., no thiaminase activity) for both methods. For the HPLC experiment, samples incubated with SRM 1946 extract were compared to those incubated with whole rainbow smelt extract and the resulting chromatograms were analyzed for additional fluorescence peaks other than that of thiamine (i.e., retention time: 12.5 min). For the microplate reader experiment, samples containing SRM 1946 extract were compared to those containing whole Pacific herring extract.

#### Enzyme Kinetics Assays

To determine optimal substrate concentrations for the fluorescence-based thiaminase activity assay, we conducted two sets of enzyme-kinetics experiments using a high-activity Pacific herring extract and a 10 min incubation. In the first experiment, thiamine concentration was varied while nicotinic acid was held constant at 30 mM; in the second, nicotinic acid concentration was varied while thiamine was fixed at 100 μM. All reactions followed the fluorescence method described above, with modifications to substrate concentrations as detailed below.

For the thiamine kinetics assay, a 1 mM thiamine solution was serially diluted with ultrapure water to yield assay concentrations of 200, 100, 50, 25, 12.5, and 6.25 μM. Thiaminase activity rates were calculated at each concentration and fitted to the Michaelis–Menten model.

To evaluate the effect of nicotinic acid concentration, we used assay concentrations of 60, 50, 40, 25, 12.5, 6.25, and 0 mM nicotinic acid. Thiaminase activity rates were calculated, with any negative values set to zero (indicating no detectable activity). To determine an optimal nicotinic acid concentration for the fluorescence-based assay, these rate data were fitted to the Hill equation.

#### pH Optimum

Rainbow smelt extract thiaminase activity was assessed over a range of pH values to identify conditions yielding maximal activity. The fluorescence method was used as described above; however, nicotinic acid solutions were prepared with buffers spanning the target pH range, approximating conditions used by Zajicek et al.^40^ Buffers contained 80 mM nicotinic acid, 100 mM sodium chloride, and either 100 mM phosphate buffer (pH 2.5, 3.85, 4.44, 5.22, 5.99, 6.19, 7.33, and 7.36) or 100 mM citric acid buffer (pH 3.44, 5.35, 6.78). Thiaminase activity rates were calculated and fitted to a Gaussian model to determine the pH range of maximal enzyme activity.

### Statistical Analysis

All statistical analyses were performed in R (version 4.4.3).^41^ Enzyme-kinetics and pH-optima model fitting were performed using the nonlinear least-squares function of the base stats package.^41^ Model residuals were visually assessed to confirm goodness of fit.

For the initial plate reader trials, differences in fluorescence intensity over time were assessed using a one-way repeated measures ANOVA and the rstatix package.^42^ A *p*-value of <0.05 was considered statistically significant.

Analyses involving nondetect (left-censored) thiaminase activity values were conducted using the NADA package.^43^ For individual species with three or more detected measurements in a given assay, summary statistics (mean and standard deviation) were estimated using the regression on order statistics (ROS) method.^44,45^ To compare the 4-NTP and fluorescence-based assays for species with censored data, the correlation was evaluated using Kendall’s rank correlation test.^45,46^ For species where all measurements from both assays were above the detection limit (i.e., rainbow smelt), the relationship was assessed using standard linear regression.

#### Fluorescence Method Limit of Detection

A limit of detection (LOD) for the fluorescence-based thiaminase assay was determined using SRM 1946 as a suitable matrix blank (i.e., no thiaminase activity). Samples incubated with SRM 1946 were analyzed on three separate days with 16 replicates measured in total. For each replicate, thiamine concentrations were measured at 0 and 30 min. The absolute difference in thiamine between these time points was calculated to quantify measurement variability in the absence of thiaminase activity. The standard deviation of these absolute differences was multiplied by 1.75 (Student’s *t-*statistic for 15 degrees of freedom at 95% confidence) to establish a detection threshold. This value was converted to an apparent enzymatic rate according to eq 1. A corresponding LOD was also calculated for a 10 min incubation period.

#### Northern Bering Sea Prey Fish and Method Comparison

For the fluorescence-based assay, each fish extract was analyzed in duplicate, with each replicate consisting of paired initial and incubated samples. Each of these replicates was measured in triplicate wells; fluorescence values from the wells were averaged and an individual thiaminase activity value was determined for each replicate. The two replicate values were then averaged to yield a final activity value for each sample. The 4-NTP method for measuring thiaminase in fish tissues was performed as described by Kraft et al., with minor modifications (Supporting Information).^31^ The LOD for the 4-NTP assay was determined by measuring the variability of blank samples, the full procedure is detailed in the Supporting Information.

## Results and Discussion

### Initial Trials – HPLC and Plate Reader

A proof-of-concept experiment used HPLC paired with fluorescence detection to measure the thiamine content of samples before and after an incubation step to estimate thiaminase activity. Fluorescence chromatograms showed complete overlap of thiamine peaks at 0, 10, and 30 min incubation time points in SRM 1946, indicating no measurable destruction over time (i.e., no thiaminase activity), consistent with our expectations for lake trout (Figure 2A). In contrast, a sample containing rainbow smelt extract showed clear loss of thiamine signal over time (Figure 2B). Thiamine was the only peak observed at the given excitation and emission wavelengths (retention time: 12.5 min). No other thiamine vitamers (e.g., thiamine monophosphate, thiamine diphosphate) or interfering peaks were observed at the specified fluorescence wavelengths. Following this experiment, all studies used a fluorescence plate reader for detection, improving assay speed and throughput.

**Figure 2:**
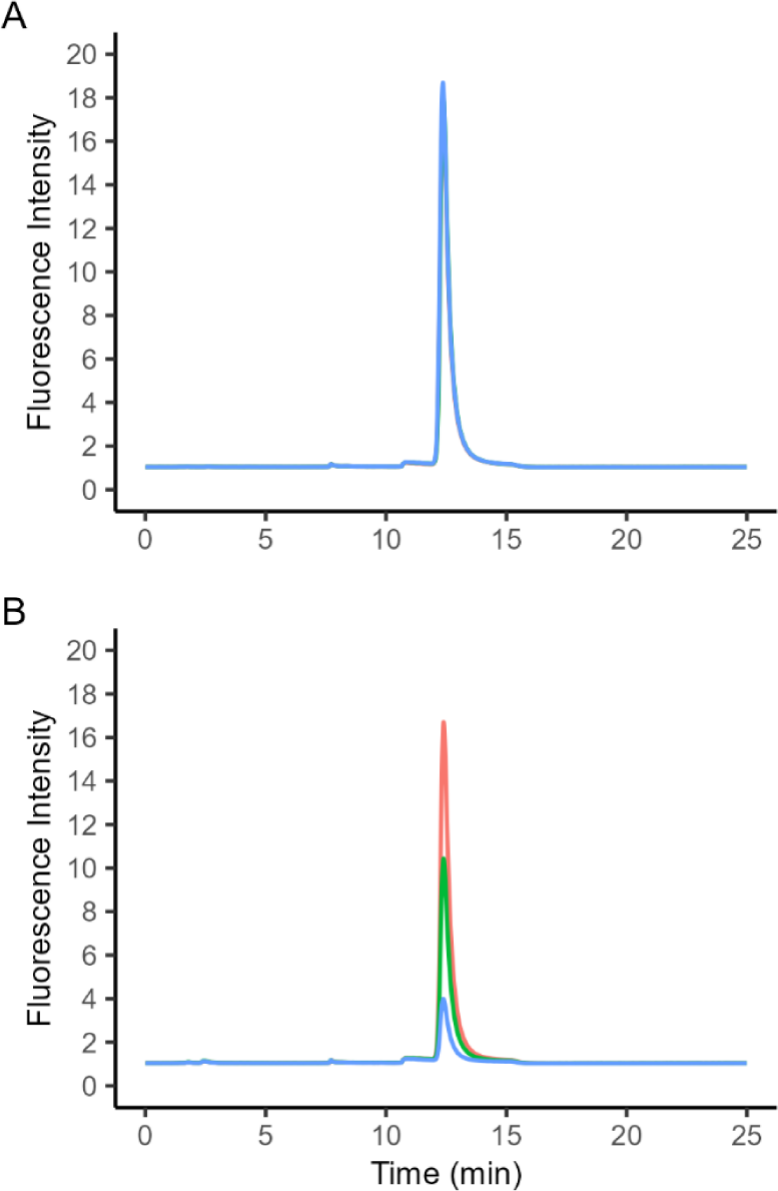
HPLC fluorescence chromatograms of added thiamine (via thiochrome; excitation: 375 nm, emission: 433 nm) to samples of (A) SRM 1946 lake trout muscle tissue and (B) whole rainbow smelt. Red, green, and blue lines correspond to incubation times of 0, 10, and 30 min, respectively. For SRM 1946, all traces overlap.

No significant thiaminase activity was detected in SRM 1946 lake trout muscle extracts, as thiochrome fluorescence intensity did not change significantly over a 30 min incubation period (one-way repeated measures ANOVA, *F*(2, 6) = 0.892, *p* = 0.458; Figure 3). In contrast, extracts from a Pacific herring showed strong evidence of thiaminase activity, with thiochrome fluorescence decreasing by an average of 71% over the 30 min incubation period. These findings prompted further experiments to optimize substrate and cosubstrate concentrations for yielding maximum activity.

**Figure 3:**
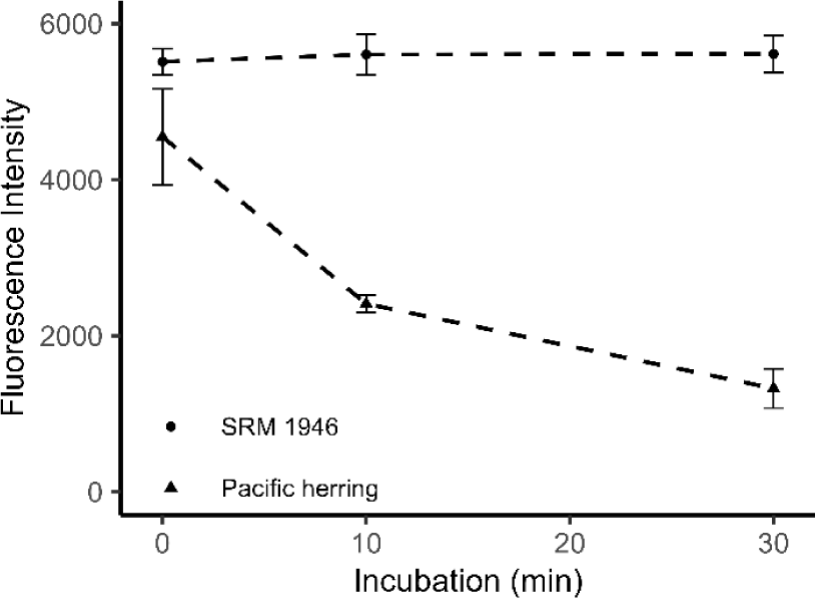
Fluorescence intensity of added thiamine (via thiochrome) in samples of SRM 1946 lake trout muscle tissue and whole Pacific herring after 0, 10, and 30 min incubations. Pacific herring represent two and SRM 1946 values represent four replicate measurements. Error bars denote ± 1 standard deviation from the mean.

### Enzyme Kinetics Assays

Thiaminase activity of a Pacific herring extract increased with rising thiamine concentration and was well described by a Michaelis–Menten model (pseudo *R* ^2^ = 0.98; Figure 4A). The fitted curve yielded a maximum reaction velocity (*V*_max_) of 13.79 ± 0.73 nmol T g^–1^ min^–1^ (*p* < 0.001) and a Michaelis constant (*K*_m_) of 27.45 ± 4.54 μM thiamine (*p* = 0.004), indicating rapid saturation at relatively low thiamine concentrations. These results confirmed that the 100 μM thiamine concentration used in the fluorescence-based assay is saturating and not rate-limiting.

**Figure 4:**
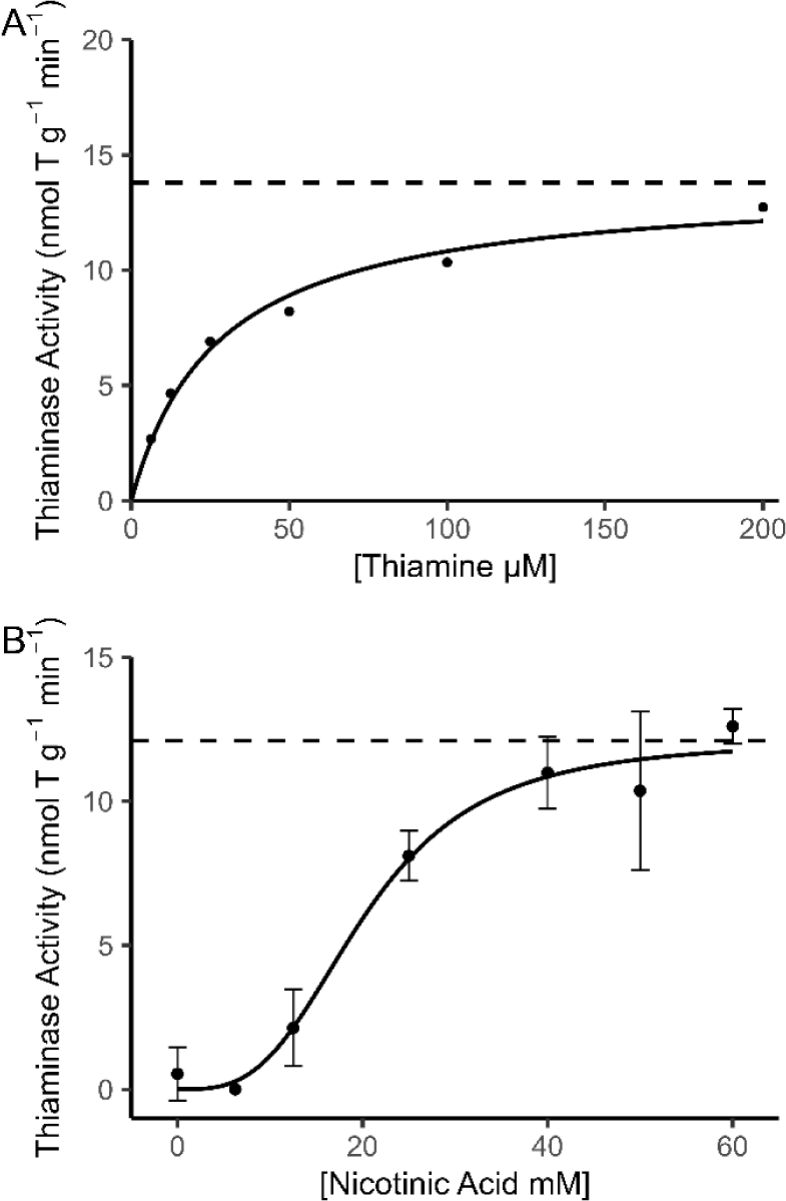
Thiaminase activity in Pacific herring extract under varying substrate conditions. (A) Michaelis–Menten kinetics observed with increasing thiamine concentrations. (B) Sigmoidal response to increasing nicotinic acid concentrations. Dashed lines indicate modeled maximum reaction velocities (*V*_max_). Error bars represent ± 1 standard deviation from the mean.

For nicotinic acid, thiaminase activity increased in a sigmoidal fashion with concentration (Figure 4B). A standard Michaelis–Menten model did not fit these data, yielding nonsignificant parameters for *V*_max_ (*p* = 0.293) and *K*_m_ (*p* = 0.430). In contrast, the Hill equation provided a substantially better description (pseudo *R* ^2^ = 0.99). The Hill fit returned a *V*_max_ of 12.10 ± 0.83 nmol T g^–1^ min^–1^ (*p* < 0.001), a half-saturation constant (*K*_half_) of 20.27 ± 1.98 mM (*p* < 0.001), and a Hill coefficient (*n*) of 3.18 ± 0.77 (*p* = 0.015), indicating positive cooperativity in nicotinic acid binding. Based on these results, a concentration of 40 mM nicotinic acid was selected for use in the assay.

The *K*_m_ for thiamine measured here with Pacific herring thiaminase falls between previously reported values from other fishes, including alewife (4.63 ± 0.64 μM) and common carp (*Cyprinus carpio* *;* 147 μM).^33,47^ Discovering positive cooperativity in thiaminase activity with respect to the cosubstrate nicotinic acid may have significant implications. Future investigations into this cooperative behavior, as well as the concentrations of naturally occurring cosubstrates in the gut of consumers (e.g., Chinook salmon), may clarify the biological significance of thiaminase in dietary prey.

### pH Optimum

The pH optimum for thiaminase activity in rainbow smelt was successfully measured using the fluorescence-based thiaminase assay (Figure 5). The resulting pH-activity profile is typical to earlier radiometric reports; activity was strongly dependent on pH, ranged multiple orders of magnitude and had a narrow range of maximum activity under mildly acidic conditions.^40^ The fitted Gaussian model (pseudo *R* ^2^ = 0.94) indicated a peak activity of 234.3 ± 14.8 nmol T g^–1^ min^–1^ (*p* < 0.001) at an optimum pH of 5.06 ± 0.06 (*p* < 0.001).

**Figure 5:**
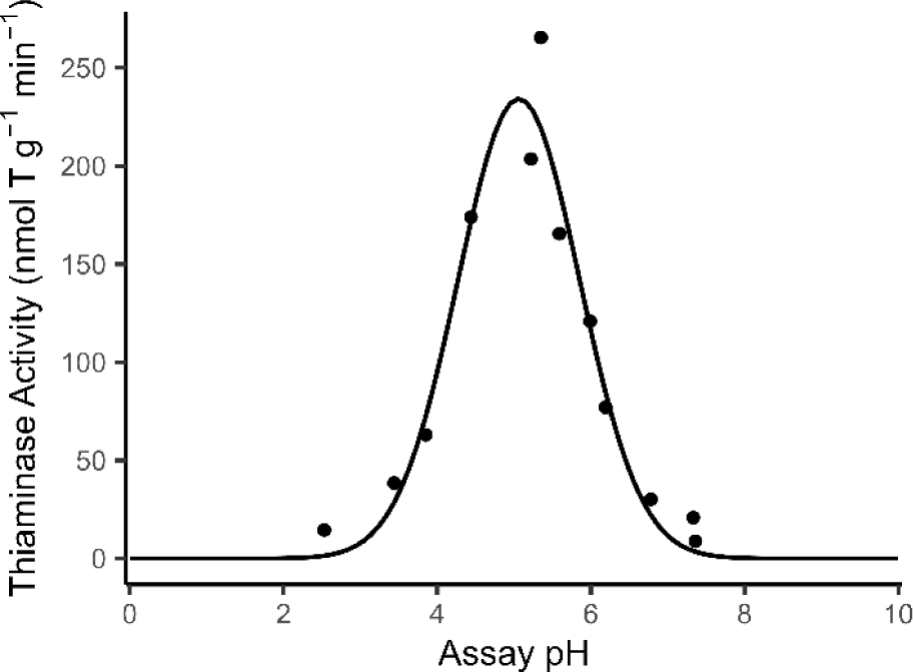
Thiaminase activity of rainbow smelt extract across a pH gradient fitted to a Gaussian model.

This optimum is slightly more acidic than the pH 5.8 reported for freshwater rainbow smelt.^40^ This discrepancy could be attributed to differences in sample collection, storage, measurement techniques, or even physiological differences between marine and freshwater populations. This study provides proof-of-concept that pH optima can be measured using this assay, but further investigation is required to draw conclusions about population-level differences.

### Method Precision and LOD

To assess the precision of the fluorescence-based thiaminase assay, 16 replicates of SRM 1946 were measured over three separate days. We calculated the detection threshold to be a decrease of 0.289 nmol thiamine between initial and incubated samples. This threshold corresponds to an enzymatic rate LOD of 2.89 nmol T g^–1^ min^–1^ for 10 min incubations and 0.965 nmol T g^–1^ min^–1^ for 30 min incubations.

A LOD of 3.63 nmol T g^–1^ min^–1^ was established for the 4-NTP assay by measuring the variation in signal of blank samples, as detailed in the Supporting Information.

Across all northern Bering Sea prey fish samples (*n* = 77), initial time point thiamine measurements deviated slightly from the nominal amount added (i.e., 10 nmol), potentially due to factors such as fluorescence quenching, coprecipitation with proteins, or other matrix effects. Initial thiamine amounts had a mean value of 9.6 ± 0.7 nmol. The relative percent difference of the initial thiamine amounts between replicates was 3.0 ± 2.1% indicating high precision.

### Method Comparison of Northern Bering Sea Prey Fish

Common prey fishes from the northern Bering Sea were analyzed for thiaminase activity using both the fluorescence-based assay and the 4-NTP assay (Table 1). Rainbow smelt exhibited the highest activity of all species tested: all specimens (*n* = 17) yielded detectable activity in both assays. The mean activity measured by the fluorescence-based assay was 147.9 ± 114.1 nmol T g^–1^ min^–1^, compared to 134.9 ± 64.9 nmol T g^–1^ min^–1^ by the 4-NTP assay. A significant linear relationship was observed between the two assays for rainbow smelt (*p* < 0.001, adjusted *R* ^2^ = 0.78, *n* = 17; Figure 6).

**Table 1:** Comparison of Thiaminase Activity using Fluorescence and 4-NTP Assays

	Fluorescence			4-NTP		
Species	Detects (*n*)^a^	Mean Rate^b^	SD^c^	Detects (*n*)^a^	Mean Rate^b^	SD^c^
Rainbow smelt	17 (17)	147.9	114.1	17 (17)	134.9	64.9
Pacific herring^d^	15 (15)	6.5	3.2	8 (15)	4.9	0.9
Capelin^d^	8 (12)	2.9	1.8	10 (12)	7.0	3.3
Sand lance	2 (11)	N/C^e^	N/C	0 (11)	N/C	N/C
Saffron cod	1 (14)	N/C	N/C	1 (14)	N/C	N/C
Walleye pollock	1 (8)	N/C	N/C	2 (8)	N/C	N/C

a Number of samples with detectable thiaminase activity out of total number of samples (*n*).

b Mean enzymatic rate (nmol thiamine destroyed per gram per minute).

c One standard deviation of enzymatic rates.

d Regression on order statistics were used to estimate mean enzymatic rates and standard deviations for groups with three or more detectable thiaminase activity measurements.

e N/C = Not calculated due to fewer than three detected values.

**Figure 6:**
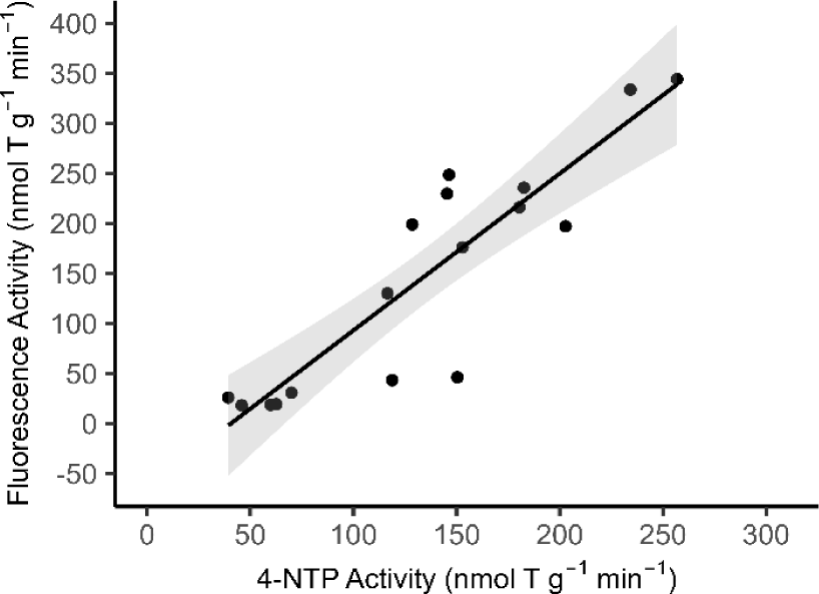
Comparison of thiaminase activity measurements between the 4-NTP and fluorescence-based assays in rainbow smelt. A significant linear relationship was observed (fluorescence-based activity = 1.6 × [4-NTP activity] – 63.5, *p* < 0.001, adjusted *R* ^2^ = 0.78). Shaded regions represent 95% confidence intervals of the fitted linear model.

Pacific herring demonstrated the second-highest mean activity in the fluorescence-based assay, averaging 6.5 ± 3.2 nmol T g^–1^ min^–1^, with all 15 samples detected. In contrast, only 8 of 15 samples were detectable by the 4-NTP assay, and the ROS estimated mean was 4.9 ± 0.9 nmol T g^–1^ min^–1^. Capelin also displayed relatively low activity, with ROS estimated means of 2.9 ± 1.8 nmol T g^–1^ min^–1^ (8 of 12 detects) for the fluorescence-based assay and 7.0 ± 3.3 nmol T g^–1^ min^–1^ (10 of 12 detects) for the 4-NTP assay. Due to the presence of nondetects in these species, the relationship between the two methods was evaluated using Kendall’s rank correlation test; no significant correlation was found for either Pacific herring (τ = 0.038, *p* = 0.13, *n* = 15) or capelin (τ = 0.29, *p* = 0.18, *n* = 12).

Previous reported radiometric measurements of freshwater rainbow smelt reported mean thiaminase activities of 2.64 nmol T g^–1^ min^–1^ (*n* = 120), and 9.24 nmol T g^–1^ min^–1^ (*n* = 32), values much lower than those measured here.^48,49^ Conversely, freshwater rainbow smelt activity measured using the 4-NTP method was much higher at 129 nmol T g^–1^ min^–1^, similar to our measurements in marine rainbow smelt.^31^ These results demonstrate the wide variability in thiaminase activity among individuals of the same species, and underscore the need for assessment of individuals across ecosystems and years.

Intermethod comparison of thiaminase activity is complicated by differences in cosubstrate (nicotinic acid vs 4-NTP) and assay pH (4.4 vs ∼6.9). Despite this, rainbow smelt showed strongly correlated activities between methods, suggesting that the optimal pH for enzyme activity may vary between cosubstrates. Capelin and Pacific herring exhibited lower activities with nonsignificant method correlations. Several capelin samples (4 of 12) fell below the detection limit for the fluorescence-based assay, whereas many Pacific herring samples (7 of 15) were nondetectable using the 4-NTP assay. These nonsignificant correlations likely reflect measurements near detection limits and potential species-specific differences in enzyme affinity for cosubstrates and sensitivity to pH conditions. These inherent interspecies enzymatic differences must be carefully considered in comparative studies. Importantly, the fluorescence-based assay developed here provides a tool to explore these complex interactions.

Thiaminase activity was very low or nondetectable in other forage fish species surveyed. For Alaska walleye pollock (*n* = 8), only one individual had activity detected (1.3 nmol T g^–1^ min^–1^) by the fluorescence-based assay, while the 4-NTP assay detected low activity in two samples (4.9 and 5.1 nmol T g^–1^ min^–1^). Conversely, for sand lance (*n* = 11) two samples had detectable activity (1.8 and 1.9 nmol T g^–1^ min^–1^) with the fluorescence-based assay, while none were detected using the 4-NTP method. One saffron cod sample (*n* = 14) was detectable (1.0 nmol T g^–1^ min^–1^) using the fluorescence-based assay and one different sample had detectable activity with the 4-NTP assay (5.4 nmol T g^–1^ min^–1^). Though rainbow smelt, Pacific herring, and capelin have been previously identified as thiaminase-containing species, this study provides the first examination of thiaminase activity in these other species.^14,50^

## Conclusions

Investigating causes of thiamine deficiency requires in-depth studies of the diets of afflicted consumers. There are numerous documented cases of thiamine deficiency occurring in animals, both in captivity and in the wild, due to consumption of raw fish rich in thiaminase. To better enable investigations of this issue, we developed a fluorescence-based microplate assay to measure thiaminase activity in extracts from whole fish. We show ability to characterize common enzyme properties, such as Michaelis–Menten kinetics and pH optima, and compared measurements of thiaminase activity in common prey fishes in the northern Bering Sea between both the new fluorescence-based assay and the conventional 4-NTP method.

Compared to other commonly used thiaminase activity assays, the fluorescence-based assay offers experimental flexibility. The standard radiometric assay relies on radiolabeled [^14^C-thiazole]-thiamine, which limits broad use. The 4-NTP assay constrains analysis to a narrow pH range and a single cosubstrate (4-NTP). In contrast, the fluorescence-based assay allows exploration across a wider pH range and the use of different cosubstrates (and concentrations) in an approachable format. This flexibility enables studies of thiaminase activity under conditions that were previously inaccessible. Since thiaminase activity can vary by orders of magnitude across pH, future studies of pH optima may help improve understanding of ecosystem-level drivers of thiamine deficiency in consumers.

In summary, the fluorescence-based thiaminase assay described here enables researchers to investigate enzyme characteristics of fish tissue derived thiaminase under a broad range of conditions. This assay allows systematic variation of temperature, thiamine and cosubstrates, and pH to conditions that may better approximate the digestive tract of consumers. Such studies may reveal mechanisms underlying thiamine deficiency in wild consumer populations and guide management strategies to mitigate its ecological impacts.

## Supplementary Material



## Notes

Additional experimental details including descriptions of the initial HPLC experiment, the 4-NTP method and LOD determination, and the fluorescence-based assay standard curve, linear range, and LOD determination (PDF)
