# Supplementary material for: Measuring Thiaminase Activity in Fish Extracts using Fluorescence Spectrophotometry
Source: ACS Meas Sci Au. 2025 Sep 19;5(6):833–41. doi: 10.1021/acsmeasuresciau.5c00091 (PMC12715735; doi:10.1021/acsmeasuresciau.5c00091)
Supplement: Supplementary file 1 [file tg5c00091_si_001.pdf]

Supporting Information

Measuring Thiaminase Activity in Fish Extracts Using Fluorescence Spectrophotometry

Drew Porter[1,2], Cody Pinger[2,*]

[1]University of Alaska Fairbanks, College of Fisheries and Ocean Sciences, 17101 Pt. Lena Loop Road, Juneau, Alaska, USA 99801

[2]NOAA, National Marine Fisheries Service, Alaska Fisheries Science Center, Auke Bay Laboratories, 17109 Pt. Lena Loop Road, Juneau, Alaska, USA 99801

*Corresponding Author: cody.pinger@noaa.gov

Initial high-performance liquid chromatography experiment

For initial experiments, samples were analyzed by high-performance liquid chromatography (HPLC) using an Agilent 1260 Infinity Quaternary LC system with fluorescence detection (excitation: 375 nm, emission: 433 nm) equipped with a Hamilton PRP1 analytical column (5 µm, 150 X 4.1 mm) and PRP-1 guard column, a flow rate of 1 mL/min and a step-wise elution described by Brown *et al.* with minor modifications.[1]

Fish samples were prepared and extracted identically to the fluorescence-based thiaminase method described in the main text. Reaction conditions were similar to the fluorescence-based method other than: a 40 mM nicotinic acid solution was used during incubations, each sample was split into replicates that were incubated for 0, 10, or 30 min, and no dilution with ultrapure water occurred. Resulting supernatants (750 µL) were washed three times with an equal volume of ethyl acetate:hexane (3:2, v:v). For derivatization, the washed supernatant (212 µL) was then transferred to a microcentrifuge tube containing 3 mM potassium ferricyanide (15 µL) and 1 M sodium hydroxide (22 µL). The resulting solutions were then transferred to clear 9 mm glass autosampler vials fitted with septa caps (Fisher Scientific) and analyzed by HPLC. Resulting thiamine fluorescence peaks (retention time: 12.5 min) were integrated and compared to external thiamine standards. The standards were prepared to originally contain 0, 1.25, 2.5, 5, and 10 nmol thiamine in ultrapure water and were derivatized and analyzed along with the samples.

4-nitrothiophenol method

The 4-nitrothiophenol (4-NTP) method followed Kraft *et al.* with minor modifications.[2] Fish extracts were prepared as described for the fluorescence assay and the same 50 mM phosphate buffer was used.

A tris(2-carboxyethyl) phosphine hydrochloride (TCEP) buffer (10 mM TCEP, 58 mM phosphate, 100 mM NaCl; pH adjusted 6.9 at room temperature) was prepared and degassed by bubbling with helium in an ice bath for 15 minutes. A concentrated 4-NTP solution (3 mg/mL dimethyl sulfoxide) was diluted into two working assay solutions: (1) 250 µM 4-NTP in TCEP buffer (control), and (2) 250 µM 4-NTP with 412 µM thiamine HCl in TCEP buffer (experimental).

Reactions were performed in clear, nonbinding 96-well microplates (Corning). Each plate included 22 unknown samples and 2 quality control sample. Wells were preloaded with 97 µL of either the control or experimental assay solution. For each sample, 3 µL of fish extract was added to two control wells and two experimental wells, resulting in four replicate wells per sample. A quality control sample (rainbow smelt extract) was included in each run at a fixed plate position to monitor inter-assay consistency.

Plates were loaded into a BioTek Synergy H1 Hybrid plate reader preheated to 37°C, and absorbance was measured at 411 nm every minute for 30 minutes.

Absorbance values were processed in Microsoft Excel.[3] Duplicate wells were averaged at each time point, and net thiaminase activity was calculated as the difference between the control and experimental absorbance values. For each 10-minute interval, the rate of change in absorbance was determined by linear regression.

Thiaminase activity (nmol g^{-1} min^{-1}) was calculated from the slope of the absorbance change using the extinction coefficient of 4-NTP in TCEP buffer (13,650 M^{-1} cm^{-1}).[4] Briefly, the slope (ΔAbs/min) was converted to mol cm^{-1} L^{-1} min^{-1} using the extinction coefficient. This value was then converted to mol g^{-1} min^{-1} by accounting for the optical path length (0.3 cm), assay volume (0.0001 L), and tissue mass per well (0.0012 g). Finally, values were converted to nmol g^{-1} min^{-1} by multiplying by 10^9.

Absorbance curves were plotted for each sample to identify anomalies. Samples showing aberrant spikes or nonlinear behavior were reanalyzed. The maximum slope observed across any 10-minute segment was recorded as the thiaminase activity for each sample.

4-nitrothiophenol method limit of detection

A method limit of detection (LOD) for the 4-NTP method was determined by analyzing replicate blank samples (n = 15). The assay was performed as described with 3 µL of 50 mM phosphate buffer substituted for fish extract. A thiaminase activity value was calculated for each blank replicate, the standard deviation of these values was determined and multiplied by the Student's t-statistic for 14 degrees of freedom at 95% confidence (1.76) to determine a LOD of 3.6 nmol g^{-1} min^{-1}.

Fluorescence-based assay standard curve, linear range, and limit of detection

Thiamine was quantified using a five-point standard curve that was run on each sample plate. Calibration standards were prepared from a 1mM thiamine HCl stock solution. The concentrations for the standard curve were chosen to span the expected range of thiamine in the thiaminase assay. A top standard of 100 µM, matching the initial thiamine concentration in the reaction mixture, was prepared and then serially diluted (1:2) to yield standards of 50, 25, and 12.5 µM. A buffer blank served as the zero point. The standards were treated similarly to samples, 25 µL of each standard added to 25 µL of phosphate buffer and 50 µL of 80 mM nicotinic acid. These solutions were then mixed with 800 µL of 3% trichloroacetic acid; 200 µL of the resulting solution was subsequently diluted with 800 µL of ultrapure water. Finally, a 425 µL aliquot of this final dilution was combined with 45 µL of 1 M sodium hydroxide and 30 µL of 3 mM potassium ferricyanide to convert thiamine into fluorescent thiochrome. A typical standard curve is presented in Figure S1.

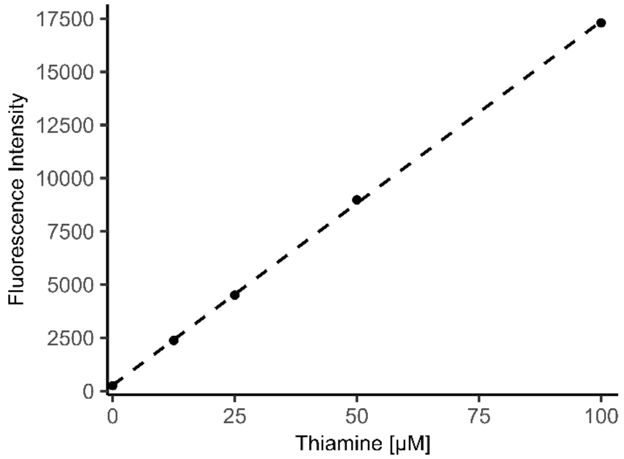

Figure S1. A representative thiamine calibration curve. The linear regression (dashed line) yielded a slope of 170.9, a y-intercept of 275.5, and a R^2 of 0.9998.

The assay's linear range and limit of detection (LOD) were also characterized. To determine the linear range a separate dilution series spanning from 0 to 6,400 µM thiamine was analyzed. From this, the assay was determined to be linear up to 800 µM thiamine. The LOD was determined as described by Thomsen *et al*. as 0.02 nmol of thiamine.[5]

SI References

(1) Brown, S. B.; Honeyfield, D. C.; Vandenbyllaardt, L. Thiamine Analysis in Fish Tissues. *Early Life Stage Mortality Syndrome in Fishes of the Great Lakes and Baltic Sea, American Fisheries Society Symposium 21*; McDonald, G., Fitzsimons, J. D., Honeyfield, D. C., Eds.; American Fisheries Society: Bethesda, Maryland, 1998; pp 73–81. DOI: 10.47886/9781888569087.ch8

(2) Kraft, C. E.; Gordon, E. R. L.; Angert, E. R. A rapid method for assaying thiaminase I activity in diverse biological samples. *PLoS ONE* **2014**, *9* (3), e92688. DOI: 10.1371/journal.pone.0092688

(3) Microsoft. *Excel 2019*, Ver. 1808. https://www.microsoft.com/en-us/microsoft-365/excel/

(4) Hanes, J. W.; Kraft, C. E.; Begley, T. P. An assay for thiaminase I in complex biological samples. *Anal. Biochem.* **2007**, *368* (1), 33–38. DOI: 10.1016/j.ab.2007.06.001

(5) Thomsen, V.; Schatzlein, D.; Mercuro, D. Limits of detection in spectroscopy. *Spectroscopy* **2003**, *18* (12), 112–114
